# Optimization of Reaction Conditions to Control Physicochemical Properties of Octenyl Succinic Anhydride Modified Normal Maize Starch

**DOI:** 10.3390/foods15111914

**Published:** 2026-05-28

**Authors:** Jiawei Gu, Xinyu Zhang, Claudia Mónika Haros, Mengting Ma, Harold Corke, Zhongquan Sui

**Affiliations:** 1 Department of Food Science & Technology, School of Agriculture and Biology, Shanghai Jiao Tong University, Shanghai 200240, China; gjw030128@sjtu.edu.cn (J.G.); zhangxinyu0729@sjtu.edu.cn (X.Z.); mmt9497@sjtu.edu.cn (M.M.); 2 Institute of Agrochemistry and Food Technology (IATA-CSIC), C/Agustín Escardino Benlloch 7, 46980 Paterna, Spain; cmharos@iata.csic.es; 3 Department of Biotechnology and Food Engineering, Guangdong Technion-Israel Institute of Technology, Shantou 515063, China; harold.corke@gtiit.edu.cn; 4 Faculty of Biotechnology and Food Engineering, Technion-Israel Institute of Technology, Haifa 3200003, Israel

**Keywords:** pasting properties, thermal properties, rheology, response surface methodology (RSM)

## Abstract

This study investigated the optimization of the physicochemical properties of normal maize starch (NMS) by esterification with octenyl succinic anhydride (OSA). The synergistic effects of OSA addition time, temperature, and total reaction time on the degree of substitution (DS) and the physicochemical properties of starch were evaluated using a combination of single-factor experiments and Response Surface Methodology (RSM). FT-IR spectroscopy confirmed the successful incorporation of OSA groups into starch molecules, with the appearance of two characteristic absorption peaks at 1724 and 1572 cm^−1^. Single-factor experiments revealed that a temperature of 40 °C, an OSA addition time of 2 h, and a total reaction time of 6 h effectively maximized the DS. These conditions balanced efficient esterification with the suppression of OSA hydrolysis and droplet aggregation. The resulting optimized OSA starch displayed lower gelatinization temperature and enthalpy, higher viscosity, more pronounced shear-thinning behavior, and greater resistance to retrogradation. Pearson correlation and simple linear regression analyses demonstrated that DS was negatively correlated with both Δ*H* and *G*′_max_. The RSM model accurately predicted the optimal synthesis parameters (41.96 °C, 2.25 h OSA addition time, 6.9 h total reaction time), achieving a validated DS of 0.0258. This study provides valuable insights for producing starch-based additives in complex food systems.

## 1. Introduction

Native starches have numerous applications in the food industry, prized for their abundance, cost-effectiveness, and excellent biodegradability and biocompatibility [1]. However, the use of native starch in complex food systems is often hampered by inherent physicochemical drawbacks, particularly its strong hydrophilicity, resulting in inferior emulsifying properties [2] and instability in challenging environmental conditions [3]. To overcome these limitations, chemical modifications, such as esterification with octenyl succinic anhydride (OSA), are widely used. This process provides the starch with necessary amphiphilic characteristics by covalently grafting hydrophobic octenyl groups onto the hydrophilic backbone [4]. This structural enhancement significantly boosts the starch’s interfacial activity, making it an effective solid particulate emulsifier. It is used to stabilize the oil-water interface in colloidal systems, such as ultrastable Pickering emulsions, high internal phase emulsions (HIPEs) [5,6,7], and oleogels [8,9]. Based on these robust systems, the utility of OSA starch has been expanded into distinct functional categories. In nutritional and delivery applications, it is widely used for fat substitution [8,9] and bioactive compound delivery [10]. In food processing, it shows great potential in food 3D printing [11] and the development of functional food packaging films [12].

The degree of substitution (DS), defined as the average number of OSA groups grafted per anhydroglucose unit [13], serves as a core indicator of modification efficiency. Higher DS values generally correlate with superior functional properties. However, enhancing DS in practical synthesis faces dual constraints: first, strict FDA food safety regulations limit OSA addition to ≤3% (*v*/*w*, based on starch dry weight) [14]. Since the DS typically increases with the amount of OSA added, this regulatory constraint inherently imposes a practical limitation on the achievable DS; second, the process is restricted by the inherent nature of the heterogeneous liquid–solid reaction, where hydrophobic OSA droplets face significant mass transfer resistance and competition from hydrolysis side reactions in the aqueous phase [13]. Despite the extensive application of Response Surface Methodology (RSM) in optimizing OSA modification parameters (pH, temperature, reaction time, and starch slurry concentration) for various botanical starches [15,16,17], most studies have typically added the OSA reagent to the starch slurry over a short period (10–30 min). However, Shang et al. [4,18] demonstrated that a continuous addition strategy—adding OSA dropwise to the starch slurry, readjusting pH to 8.5 after each drop, and waiting until pH stabilizes before adding the reagent, altering the substitution pattern of OSA groups and yielding superior product performance at equivalent DS levels. This finding reveals the addition strategy to be a critical process variable rather than a mere procedural step.

Nevertheless, this method requires dropwise addition with pH stabilization between successive drops, making it impractical for industrial-scale production. To date, no industrially viable continuous addition strategy has been established. Furthermore, the synergistic effects between OSA addition strategy and other process parameters remain to be systematically investigated. To address these problems, the continuous addition strategy was modified as follows: a single OSA drop was defined as 50 μL, the required number of drops was calculated from the total OSA dosage, and the total addition time was evenly divided by the number of drops to achieve timed interval addition, thereby converting the total OSA addition time into a new variable to be optimized. Based on preliminary literature review and pre-experimental results, pH, OSA dosage, and slurry concentration were adopted as fixed factors: pH was set at 8.5 to strike a balance between starch hydroxyl activation and OSA hydrolysis—below this value, esterification is limited, whereas above it, excess NaOH reacts with OSA to form sodium carboxylate, resulting in a decreased DS [19]; OSA dosage was set at 3% (*v*/*w*, based on starch dry weight) to maximize the extent of modification within regulatory limits; preliminary single-factor experiments indicated that slurry concentration (fixed at 30%) had no significant effect on DS within the operational range. On this basis, with OSA addition time, reaction temperature, and total reaction time as three independent variables, single-factor experiments were first conducted to examine the effect of each variable on the DS and to analyze the physicochemical properties of OSA-modified NMS and their correlations with DS. Subsequently, a Box–Behnken RSM model was constructed using DS as the response value to systematically investigate the individual and interactive effects of the three variables on DS, ultimately determining the optimal reaction conditions and establishing an efficient standardized synthesis protocol.

## 2. Materials and Methods

### 2.1. Materials

Normal maize starch (NMS, amylose content: 25.2% ± 0.1%) was purchased from Gaofeng Technology Co., Ltd. (Suzhou, China). Octenyl succinic anhydride (OSA, 99%, mixture of *cis* and *trans* isomers) was obtained from Beyotime Biotechnology Co., Ltd. (Shanghai, China). All other reagents used were of analytical purity and acquired from Aladdin Reagent Co., Ltd. (Shanghai, China).

### 2.2. Preparation of OSA-Modified Starch

The preparation of OSA-modified NMS was performed following the protocol described by Sui et al. [20], with slight modifications. Initially, a uniform starch slurry was obtained by suspending NMS (20 g, dry basis, db) in deionized water (45 mL) with continuous magnetic stirring. The pH of the slurry was adjusted to 8.5 with 0.1 M NaOH, followed by incubation at 40 °C for 10 min. Subsequently, 0.6 mL of OSA (3%, *v*/*w*, based on starch dry weight) was introduced into the system in twelve 50 μL increments over a period of 2 h. The esterification reaction proceeded for a total of 6 h, during which the pH was held constant at 8.5 via the automated addition of 0.1 M NaOH. Reaction termination was achieved by neutralizing the slurry to pH 6.5 with 0.1 M HCl. The solid product was recovered through centrifugation, washed twice with deionized water and finally with anhydrous ethanol, and then oven-dried at 37 °C for 24 h. Finally, the dried sample was pulverized, passed through a 100-mesh screen, and kept in a cool, dry environment. A control sample prepared under the same alkaline conditions but without OSA addition showed negligible differences from native NMS; therefore, native NMS was used as a reference throughout.

### 2.3. Single Factor Experiments

Single-factor experiments were conducted to prepare various modified starch samples by investigating three independent variables: temperature (0, 25, 35, 40, and 45 °C; the 0 °C condition was maintained using an ice-water bath), OSA addition time (0.5, 1.0, 1.5, 2.0, and 2.5 h), and total reaction time (2, 4, 6, and 8 h).

### 2.4. DS of OSA-Modified Starch

OSA-modified starch (5 g, db) was accurately weighed and placed into a 150 mL beaker, then approximately 5 mL of isopropanol was added and mixed. A 21% (*v*/*v*) HCl-isopropanol solution (25 mL) was added to rinse any sample adhering to the beaker walls, followed by magnetic stirring for 30 min. Subsequently, 100 mL of 95% isopropanol solution was added, and the mixture was stirred for an additional 10 min. The solution was filtered through a Büchner funnel. The residue was washed with 95% isopropanol solution until the filtrate was free of chloride ions (tested using 0.1 M silver nitrate solution). The residue was then transferred into a 600 mL beaker. The Büchner funnel was carefully rinsed with 95% isopropanol solution, and the washings were combined into the beaker. Water was added to bring the total volume to 300 mL. The mixture was heated in a boiling water bath with stirring for 10 min. After adding 3 drops of phenolphthalein indicator, the solution was titrated while hot with 0.1 M NaOH solution until a pink color persisted for 30 s, indicating the endpoint.

The DS of OSA-modified starch was expressed as:

(1) $$DS_{\mathrm{OSA}}=\frac{162\;\times \;M\;\times \;(\;A\;-{\;A}_{0}\;)}{1000\;\times \;W-210\;\times \;M\;\times \;(\;A-A_{0}\;)}$$

where 162 = molecular weight of a glucosyl unit; 210 = molecular weight of an octenyl succinyl group; *A* = the volume of 0.1 M NaOH consumed by the OSA starch; *A*_0_ = the volume of 0.1 M NaOH consumed by the native starch blank; *M* = molarity of 0.1 M NaOH solution; and *W* = dry weight of starch.

### 2.5. Fourier Transform Infrared Spectroscopy (FT-IR)

The sample was mixed with KBr at a ratio of 1:100 (*w/w*), pressed into a transparent pellet, and analyzed by FT-IR (Nicolet 6700, Thermo Fisher Scientific, Waltham, MA, USA) to characterize its chemical structure. The wavenumber scanning range was 4000–400 cm^−1^, with 16 scans and a resolution of 4 cm^−1^ [21].

### 2.6. Determination of Pasting Properties

The pasting properties of the starch samples were determined using a Rapid Visco Analyser (RVA 4500, Perten Instruments, Hägersten, Sweden). Starch samples (1.4 g, db) were dispersed in deionized water within the RVA canister to form a total weight of 28 g. The analysis followed a standard heating and cooling cycle: the slurry was first equilibrated at 50 °C for 1 min, ramped up to 95 °C within 3.42 min, maintained at 95 °C for 2.30 min, cooled back to 50 °C within 3.48 min, and finally held at 50 °C for 2 min. Regarding agitation, the paddle rotated at 960 rpm for the initial 10 s to ensure homogenization, after which the speed was decreased to a constant 160 rpm for the rest of the assay.

### 2.7. Determination of Thermal Properties

Thermal properties of the starch samples were assessed utilizing a differential scanning calorimeter (Q2000, TA Instruments, New Castle, DE, USA). The starch sample (2 mg, db) was mixed with deionized water (6 μL) and hermetically sealed within aluminum DSC pans. These pans were allowed to equilibrate at ambient temperature overnight. The scanning process covered a range from 30 to 130 °C with a constant heating rate of 10 °C/min. Final data processing was carried out via TRIOS Software 5.1.1 (TA Instruments, New Castle, DE, USA).

### 2.8. Determination of Steady Shear Rheological Properties

Steady shear rheological properties were evaluated using an AR1000-N rheometer (TA Instruments, Wilmington, DE, USA). The instrument was configured with a 40 mm parallel plate geometry, and the gap was set to 1 mm. Freshly prepared starch pastes from the RVA analysis were transferred to the rheometer plate. To prevent moisture loss, the exposed edges of the sample were coated with a thin layer of low-density silicone oil. The samples were equilibrated at 25 °C for 5 min prior to measurement.

The obtained flow data were modeled using the Power Law equation:

(2) $$\sigma \;=\;K{\dot{\gamma }}^{n}$$

where *σ* is the shear stress (Pa), *K* is the consistency coefficient (Pa·s^n^), $\dot{\gamma }$ is the shear rate (s^−1^), and *n* is the dimensionless flow behavior index.

### 2.9. Determination of Dynamic Rheological Properties

Dynamic rheological tests were conducted using starch suspensions with a concentration of 20% (*w*/*w*). Preparation involved dispersing the starch powder in deionized water and stirring magnetically for 30 min at 25 °C. The samples were then placed on a rheometer fitted with 40 mm parallel plates and a gap setting of 1 mm. The testing protocol consisted of a heating and cooling cycle (25–95–25 °C) carried out at a constant temperature gradient of 2 °C/min.

### 2.10. Correlation and Linear Regression Analysis

Pearson correlation analysis was performed to evaluate the linear relationships between DS and the physicochemical properties (PV, FV, Δ*H*, and *G*′_max_) of OSA-modified NMS using data from all single-factor experimental conditions (*n* = 12). Simple linear regression models (*Y* = *a*·DS + *b*) were subsequently fitted for each dependent variable (PV, FV, Δ*H*, and *G*′_max_) against DS as the independent variable. The coefficient of determination (*R*^2^) and the level of significance (*p*) were calculated for each regression to assess the goodness of fit.

### 2.11. Experimental Design for Optimization

#### 2.11.1. Response Surface Methodology (RSM)

Optimization was executed using Design-Expert software (v. 13.0.1.0, Stat-Ease Inc., Minneapolis, MN, USA). RSM was employed to optimize three key parameters of OSA starch modification: Temperature (*A*), OSA addition time (*B*), and Total reaction time (*C*), with DS as the response.

Based on the preliminary single-factor experiments used to define the suitable ranges and center points, a three-factor, three-level Box–Behnken design (BBD) was employed. This design consisted of 17 randomized runs, including 12 factorial points and 5 replicates at the center point, as detailed in Table 1. To determine the optimal conditions for maximizing DS, the experimental results were regressed against a polynomial model. The statistical significance of both the independent variables and their interactive effects was assessed using Analysis of Variance (ANOVA).

The experimental data were fitted to a second order polynomial model as:

(3) $$Y\;=\;a_{0}\;+\;a_{1}\;\times \;A\;+\;a_{2}\;\times \;B\;+\;a_{3}\;\times \;C\;+\;a_{12}\;\times \;\mathrm{AB}\;+\;a_{13}\;\times \;\mathrm{AC}\;+\;a_{23}\;\times \;\mathrm{BC}\;+{\;a}_{11}\;\times \;{A\;}^{2}\;+\;a_{22}\;{\;\times \;B}^{\;2}\;+\;a_{33}\;\times \;{C\;}^{2}$$

where *Y* was the response function, *a*_0_, *a*_1_, *a*_2_, *a*_3_, *a*_12_, *a*_13_, *a*_23_, *a*_11_, *a*_22_, *a*_33_ were coefficients, *A*, *B* and *C* represented the temperature, OSA addition time and total reaction time, respectively.

#### 2.11.2. Regression Analysis

Data analysis and regression modeling were executed using Design-Expert 13.0.1.0 (Stat-Ease Inc., Minneapolis, MN, USA). To identify the most suitable correlation for the experimental data, the software screened various polynomial models, ranging from linear to quartic. The statistical significance of both the overall model and specific terms was determined via *F*-values and *p*-values. Model adequacy was further examined using the coefficient of determination (*R*^2^) and the coefficient of variation (CV), while the Adeq Precision metric was utilized to gauge the signal-to-noise ratio. Based on these analyses, a regression equation was derived to predict optimal processing parameters. Finally, verification trials were performed to confirm the accuracy of the predicted values and the validity of the fitted RSM model.

### 2.12. Statistical Analysis

All experiments were performed in triplicate (*n* = 3), and the experimental data are expressed as mean ± standard deviation (SD). Statistical analysis was performed using SPSS 27.0 (IBM, Armonk, NY, USA). Data were analyzed by one-way analysis of variance (ANOVA) followed by Duncan’s multiple range test. A value of *p* < 0.05 was considered statistically significant.

## 3. Results and Discussion

### 3.1. Effects of Different Single-Factor Changes on DS

**Figure 1:**
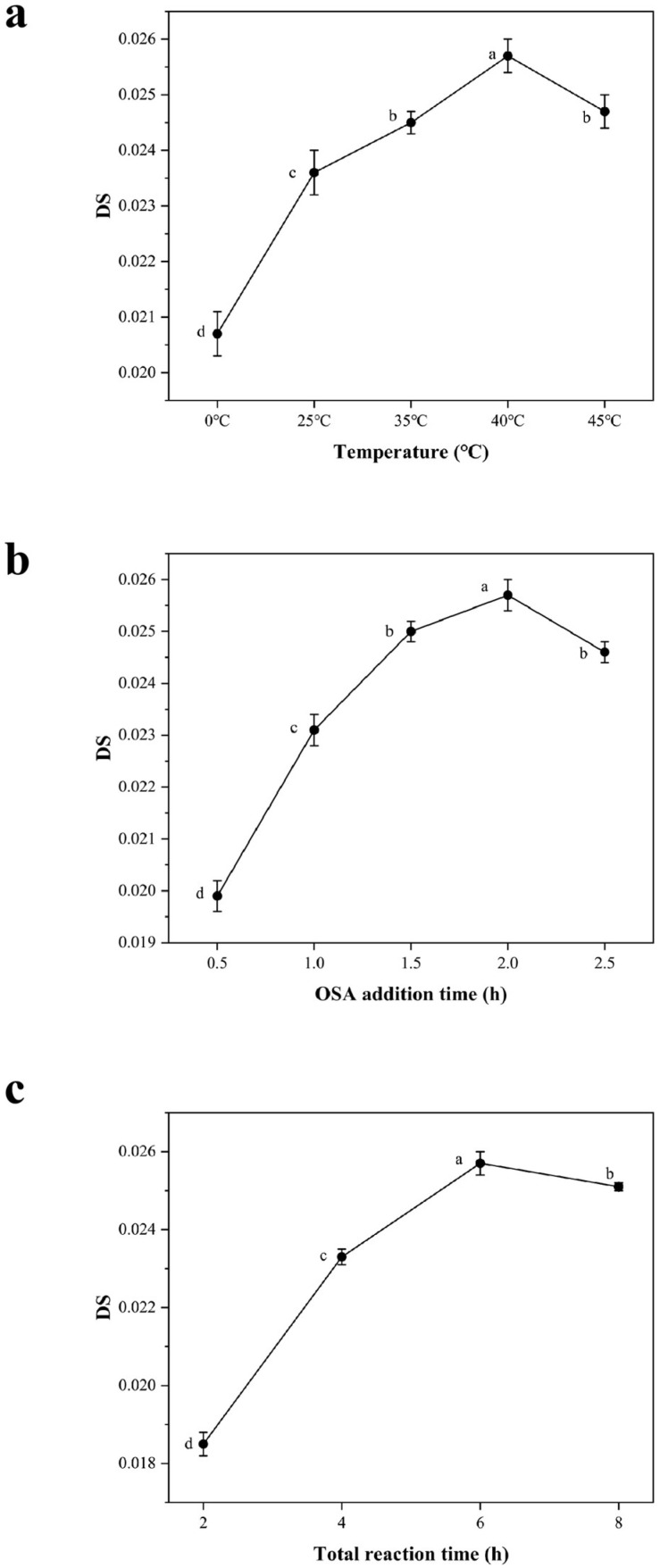
DS of OSA-modified NMS under varying single-factor conditions: (**a**) temperature; (**b**) OSA addition time; (**c**) total reaction time. Different letters (a–d) in the figure indicate significant differences (*p* < 0.05).

First, the effect of temperature on the DS is shown in Figure 1a. In the range of 0 °C to 40 °C, the DS showed an upward trend with increasing temperature. Esterification is a fundamentally endothermic process, exhibiting sluggish kinetics under low-temperature conditions. Elevating the reaction temperature enhances the thermal motion of OSA and starch molecules and increases their intermolecular collision frequency, thereby accelerating the reaction rate and promoting ester bond formation. Furthermore, a moderately elevated temperature promotes starch granule swelling and facilitates the intragranular diffusion of the OSA reagent, leading to an increased DS [22,23]. However, when the temperature increased to 45 °C, the DS decreased. Although elevated temperatures accelerate the forward esterification reaction, excessive heating may increase the aqueous solubility of OSA [24], thereby intensifying competitive hydrolysis as a side reaction and leading to premature consumption of the effective reagent [25]. Additionally, under alkaline conditions, elevated temperatures promote starch granule aggregation, which impedes OSA dispersion within the reaction system and further compromises DS [19].

Second, the effect of OSA addition time on the DS is shown in Figure 1b. Excessively rapid addition of OSA resulted in an instantaneously high concentration, which might induce OSA self-aggregation in large oil droplets. This aggregation consequently reduced the interfacial surface area available for the reaction between OSA and starch granules, while also impeding OSA diffusion in the aqueous medium and its penetration into the interior of starch granules. In contrast, moderate OSA addition time could maintain a relatively stable OSA concentration to make the reagent disperse as fine droplets and penetrate more easily into the starch granule interior, thereby facilitating a higher DS. If the OSA addition time was excessively prolonged with a constant total reaction time, the addition of OSA at later stages would not have sufficient reaction time, leading to a decrease in DS.

Finally, the effect of total reaction time on the DS is illustrated in Figure 1c. As the time extended, the DS increased significantly and reached a peak at 6 h, indicating that OSA molecules required sufficient time to penetrate into the granule interior. However, a decline in DS was observed when the reaction was prolonged to 8 h. This was attributed to the thermodynamic instability of the ester bonds: under continuous alkaline and heated conditions, the formed ester bonds undergo alkaline hydrolysis (saponification) reaction [26,27].

### 3.2. FT-IR Analysis

**Figure 2:**
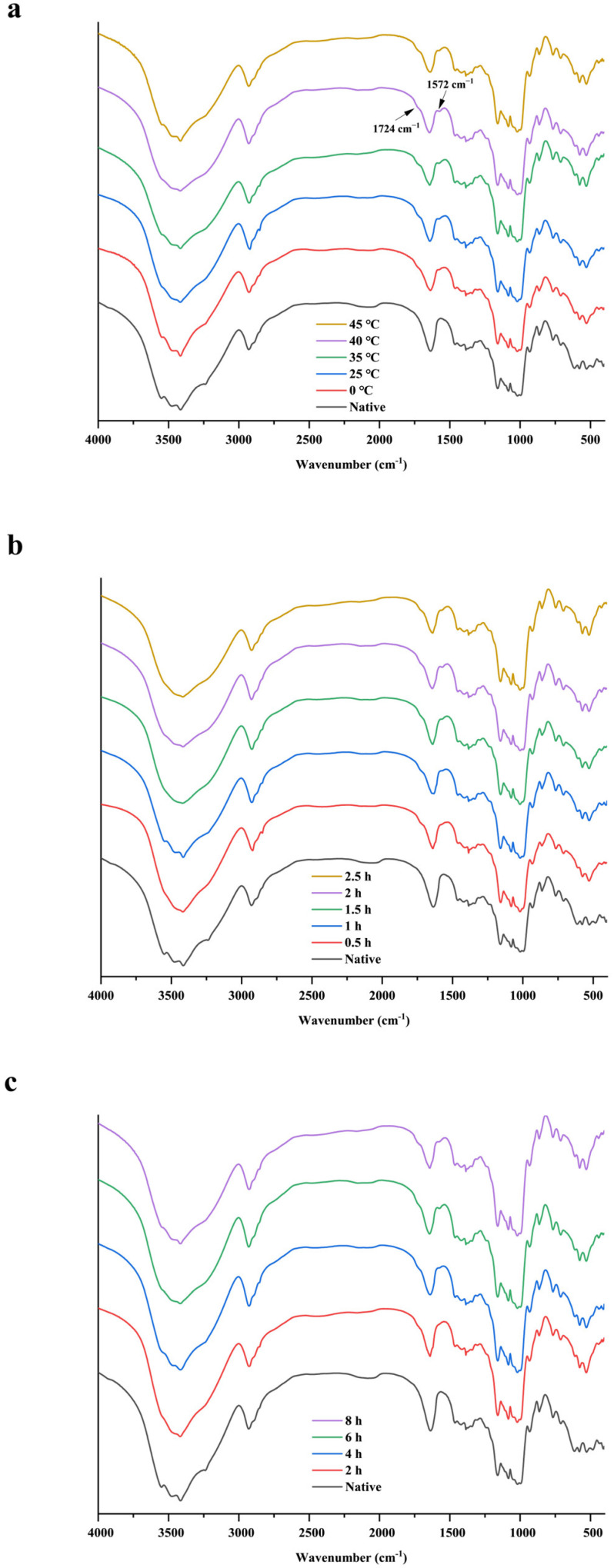
FT-IR spectra of OSA-modified NMS under varying single-factor conditions: (**a**) temperature; (**b**) OSA addition time; (**c**) total reaction time.

The OSA-modified NMS exhibited two new absorption peaks at 1724 and 1572 cm^−1^ in its FT-IR spectra, compared with native NMS (Figure 2). The peak at 1724 cm^−1^ corresponds to the stretching vibration of an ester carbonyl group (C=O), whereas the peak at 1572 cm^−1^ is attributed to the asymmetric stretching vibration of the carboxylate anion (RCOO^−^) [21]. The presence of these two characteristic peaks confirms the successful esterification and incorporation of OSA groups into the starch molecules. 

### 3.3. Effects of Different Single-Factor Changes on Pasting Properties

Table 2 illustrates the pasting characteristics of OSA-modified NMS prepared under various single-factor conditions. All OSA-modified NMS samples showed higher PV, TV, FV, and SB, compared with native NMS. The observed trend can be interpreted through several interacting mechanisms. When OSA groups replace hydroxyl groups in starch granules, they disrupt the tight hydrogen-bonding network between hydroxyl groups, leading to a looser structure [28]. The bulky OSA groups also generate substantial steric hindrance, creating additional void spaces inside the granules. Water molecules can easily penetrate into the modified granules, interacting with amylopectin and accelerating their hydration. As a result, the double-helical and crystalline structures of amylopectin dissociate more easily during heating, yielding entangled network structures [29]. After gelatinization, the OSA groups—through their considerable steric bulk and intermolecular repulsion—interfere with hydrogen-bond-mediated re-association of starch chains, keeping them in a more extended conformation [30].

Regarding the influence of temperature, the BD reached its minimum at 40 °C. The higher density of internal ester groups effectively reinforced the granule integrity, enhancing resistance to high-temperature shear. However, BD increased again while FV declined slightly at 45 °C. This trend reflected competitive hydrolysis of OSA groups at elevated temperatures, which reduced the number of internal OSA groups.

With respect to the OSA addition time, rapid introduction (0.5 h) resulted in maximum BD. Rapid addition leads to reagent aggregation and uneven substitution [31]. Consequently, the starch granules lacked uniform structural reinforcement, making them susceptible to shear breakdown. Notably, at a 2 h addition time, FV peaked and BD reached its minimum, suggesting that the optimized substitution pattern effectively reinforced the granule structure. However, prolonging addition to 2.5 h caused PV and FV to decline, mirroring the drop in DS due to insufficient reaction time for the late-added reagent.

Concerning the total reaction time, extending the duration from 2 to 6 h resulted in a marked increase in PV and FV, coupled with a significant reduction in BD. This trend corresponds to the increase in DS. When the reaction was further extended to 8 h, the viscosity parameters declined due to ester bond saponification under alkaline conditions.

### 3.4. Effects of Different Single-Factor Changes on Thermal Properties

The thermal characteristics of both native and OSA-modified NMS are summarized in Table 3. Relative to the native NMS, all OSA-modified samples exhibited significantly lower *T*_o_, *T*_p_, *T*_c_, and ∆*H*. The decrease can be attributed to the interference of OSA groups with intermolecular interactions among starch molecules, enabling the granules to swell and gelatinize at lower temperatures [32]. Furthermore, OSA groups destabilize the double-helical structures within starch, lowering the energy required for helix unwinding, which consequently manifests as a decrease in Δ*H* [33].

As the reaction temperature rose to 40 °C, *T*_o_, *T*_p_, *T*_c_, and ∆*H* decreased continuously. This trend corresponds to the increase in DS, as the moderately elevated temperature facilitated the incorporation of a greater number of OSA groups. Although the sample modified at 40 °C achieved the highest DS, its gelatinization temperatures were surprisingly lower than those of the 45 °C sample. This could be explained by the fact that sustained heating at 45 °C partially swelled the starch granules and loosened intermolecular hydrogen bonds. At this temperature, the direct thermal disruption of granule structure appeared to outweigh the effect of the slightly lower DS, leading to a reduced gelatinization temperature.

Regarding the effect of OSA addition time, extending the duration from 0.5 h to 2 h resulted in a continuous decrease in *T*_o_, *T*_p_, *T*_c_, and ∆*H*. However, extending the addition to 2.5 h caused a slight rebound in these thermal parameters. When the total reaction time was kept constant, an excessively long addition time severely shortened the effective reaction time available for the OSA added at later stages. Consequently, these late-added OSA fractions did not have adequate time to overcome the diffusion barriers to reach the starch granules, resulting in an insufficient number of OSA groups incorporated into starch molecules.

In terms of total reaction time, *T*_o_, *T*_p_, *T*_c_, and ∆*H* reached their minimum at 6 h, indicating that this duration was sufficient to allow OSA molecules to diffuse into the interior regions [34].

### 3.5. Effects of Different Single-Factor Changes on Shear Flow Properties

As shown in Table 4, the flow index (*n*) of all tested samples was significantly lower than 1. Furthermore, after modification with OSA, the consistency coefficient (*K*) of NMS increased drastically, indicating that the modified starch pastes exhibited strong pseudoplastic behavior. This phenomenon can be attributed to the introduction of hydrophobic octenyl groups. The dramatic increase in *K* reflected the promoted hydrophobic associations between starch chains, the increased hydrodynamic volume, and a more complex molecular entanglement network, thereby enhancing the structural stability of the system [32]. Further analysis of thixotropic characteristics revealed that the *K* values of the upward curves for all samples were higher than those of the downward curves, indicating that the starch pastes possessed typical thixotropy. This can be attributed to the progressive disintegration of the internal network of the starch pastes under ascending shear stress. During the subsequent decrease in shear, the rate of structural re-association significantly lagged behind the rate of disruption, resulting in the formation of a distinct hysteresis loop [35].

Both *K* and *n* reached their maximum in the modified samples at 40 °C. This enhancement can be attributed to the optimal DS achieved at this temperature. The highest number of grafted hydrophobic groups promoted stronger intermolecular associations, thereby enhancing the pasting viscosity. However, at 45 °C, the *K* value declined. This reduction is linked to the decreased DS and the potential degradation of starch chains under high-temperature alkaline conditions, which compromised the structural integrity of the fluid [36].

Extending the OSA addition time from 0.5 h to 2 h significantly increased *K* values. This mirrors the DS results, confirming that a controlled addition strategy enhances the thickening capacity of the modified starch by ensuring more effective esterification. An excessively long addition time (2.5 h) led to a slight decrease in *K*, attributed to the incomplete reaction of the late-added OSA.

Similarly, regarding the total reaction time, *K* reached its peak at 6 h. The progression of the substitution reaction disrupts the crystalline structure, facilitating the formation of a stronger network. However, extending the reaction to 8 h caused a decrease in *K* and *n*, as ester bond saponification occurred under alkaline conditions.

### 3.6. Effects of Different Single-Factor Changes on Dynamic Oscillatory Properties

Table 5 summarizes the dynamic rheological properties of NMS and OSA-modified starches prepared under different conditions, including the maximal storage modulus (*G*′_max_), loss modulus (*G*″_*G*′max_), loss tangent (tan *δ*_*G*′max_), and the temperature at maximal storage modulus (*T*_*G*′max_). During the heating stage, NMS exhibited the highest *G*′_max_ and *T*_*G*′max_, indicating that its granules maintained high structural rigidity during gelatinization. In contrast, OSA modification significantly reduced the *G*′_max_ and *T*_*G*′max_ values of all samples, a trend that was in line with the reported decrease in gelatinization temperature and changes in thermal stability of modified starches [13].

As the reaction temperature increased from 0 °C to 40 °C, *G*′_max_ gradually decreased while tan *δ*_*G*′max_ increased. The extensive introduction of OSA groups at 40 °C (highest DS) weakened the internal hydrogen bonding via steric hindrance and electrostatic repulsion. This resulted in a looser granule structure that disintegrated more easily under heating [37]. At 45 °C, *G*′_max_ rebounded slightly, correlating with the reduced effective substitution efficiency caused by accelerated hydrolysis.

The effects of OSA addition time and total reaction time followed a similar pattern. Extending the addition time to 2 h or the total reaction time to 6 h led to a significant decrease in *G*′_max_ and an increase in tan *δ*_*G*′max_. However, an excessively long reaction time (8 h) caused a rebound in modulus, potentially due to substituent instability.

During the cooling stage, as the temperature decreased, the gelatinized amylose molecules typically rearrange to re-form double helices, resulting in increased gel strength [38]. NMS showed a *G*′_25°C_ as high as 6542 Pa, indicating a strong tendency toward retrogradation. Conversely, the *G*′_25°C_ of all OSA-modified samples decreased drastically, dropping by 61.6–71.9% compared to that of native NMS, whereas tan *δ*_25°C_ was significantly higher, exhibiting an increase of 100–175.9%. This suggested that the OSA groups effectively hinder starch chain rearrangement and inhibit the retrogradation process, thereby forming a gel network with a softer texture and less elasticity but higher stability.

### 3.7. Correlation Analyses and Simple Linear Regressions

To quantitatively evaluate the influence of DS on the physicochemical properties of OSA-modified NMS, Pearson correlation analyses and simple linear regressions were performed using data from all single-factor experimental conditions (Table 6 and Table 7). DS was negatively correlated with both Δ*H* (*r* = −0.960, *p* < 0.001) and *G*′_max_ (*r* = −0.972, *p* < 0.001), corresponding to coefficients of determination (*R*^2^) of 0.922 and 0.945, respectively. In contrast, PV showed no significant correlation with DS (*r* = −0.307, *p* = 0.332). Similarly, FV displayed a positive but non-significant trend with DS (*r* = +0.503, *p* = 0.095). These results demonstrated that DS was closely related to the thermal and rheological properties, but lacked a significant relationship with the pasting properties of OSA-modified NMS.

### 3.8. Model Fitting and Adequacy Checking

Table 1 summarizes the DS values obtained under the various experimental conditions. To identify the optimal parameters for maximizing DS, a mathematical model was established based on the quadratic equation derived from the RSM analysis. The resulting regression equation, expressed in terms of coded variables, is as follows:

*Y* = −0.007833 + 0.002015* A* − 0.006265 *B* − 0.000683 *C* + 0.00031 *AB* + 0.0001 *AC*

            + 0.001 *BC* − 0.00004 *A*^2^ − 0.00319 *B*^2^ − 0.000406 *C*^2^

where *A*, *B* and *C* were the temperature, OSA addition time and total reaction time, respectively.

The fitting statistics, detailed in Table 8, provide information on model significance, lack of fit, and variable interactions. The reliability and predictive accuracy of the chosen model were rigorously confirmed by these statistical metrics. Notably, the lack of fit was statistically insignificant (*p* = 0.108 > 0.05), demonstrating that the model adequately fits the experimental data and the residual variance is minimal. Furthermore, the model’s reliability is strongly corroborated by an exceptionally high adjusted coefficient of determination (Adjusted *R*^2^ = 0.984), indicating that 98.4% of the response variability is accurately captured, and that the coefficient of variation (CV = 0.815%) is low, reflecting excellent experimental precision and reproducibility. The results indicated that temperature (*A*), OSA addition time (*B*), and total reaction time (*C*) all significantly influenced the value of DS, as evidenced by their *p*-values (*p* = 0.0184, *p* = 0.0028, and *p* < 0.0001, respectively).

Furthermore, to evaluate the relative importance of the variables and their interactions, their effect sizes (represented by the Sum of Squares) and *p*-values were analyzed. For the linear terms, total reaction time (*C*) exhibited the highest significance (*p* < 0.0001) and the largest effect size (Sum of Squares = 6.48 × 10^−6^), followed by OSA addition time (*B*; *p* = 0.0028) and temperature (*A*; *p* = 0.0184). The dominant role of the total reaction time is consistent with the kinetics of the heterogeneous esterification process. The reaction between hydrophobic OSA droplets and the hydrophilic starch matrix is inherently slow, requiring extended periods for adequate mass transfer and subsequent chemical grafting to occur. Additionally, all interaction terms (*AB*, *AC*, *BC*) and quadratic terms (*A*^2^, *B*^2^, *C*^2^) were highly significant (*p* < 0.0001). Among the interactions, *AC* and *BC* demonstrated a more pronounced effect (Sum of Squares = 4.00 × 10^−6^) compared to *AB* (Sum of Squares = 2.40 × 10^−6^). For the quadratic terms, the non-linear impacts ranked as *C*^2^ > *A*^2^ > *B*^2^, consistent with the magnitude of their respective variance contributions. These statistical metrics collectively confirm the dominant role of total reaction time (*C*) and clarify the specific contributions of each factor and their interactions to the DS.

### 3.9. Optimization of Reaction Conditions for OSA Modification

**Figure 3:**
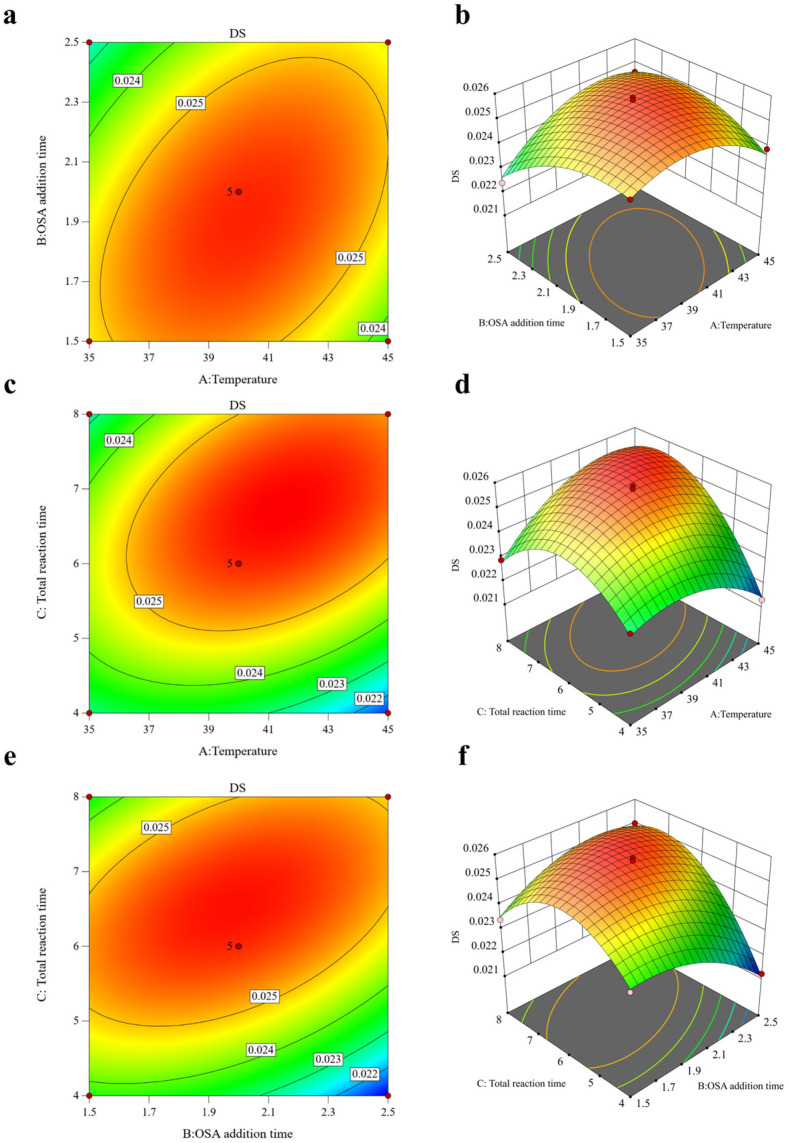
Contour plots and three-dimensional response surface plots illustrating the interactive effects of various factors on the DS: (**a**,**b**) temperature and OSA addition time; (**c**,**d**) temperature and total reaction time; (**e**,**f**) OSA addition time and total reaction time.

The optimum reaction conditions for OSA modification were determined using Box–Behnken design (BBD) response surface methodology (RSM) (Figure 3). The BBD model predicted a maximum DS at conditions near the center point of the experimental design. According to the RSM prediction, the optimal conditions were 41.96 °C for temperature, 2.25 h for OSA addition time, and 6.9 h for total reaction time, under which the model predicted a DS of 0.0259.

While the ANOVA results demonstrated the model’s statistical significance, confirmation trials were executed to corroborate its predictive precision. For operational feasibility, the processing parameters were adjusted to 42 °C, 2.3 h (OSA addition time), and 7 h (total reaction time). The observed mean DS under these settings was 0.0258, exhibiting negligible deviation from the predicted value of 0.0259. A relative standard deviation (RSD) of 0.77% highlighted the high reproducibility of the developed model.

Regarding practical applications, while the continuous addition strategy significantly enhances the DS and functional properties of OSA-modified starch, its transition to an industrial scale requires a comprehensive consideration of practical limitations. Although this approach maximizes reagent utilization and endows the final product with exceptional textural properties, the continuous feeding mechanism (lasting 2.25 h) inevitably increases process complexity, as well as time and energy costs, compared to the conventional batch method. Therefore, future industrial scale-up necessitates a rigorous cost–benefit analysis to weigh the performance advantages of high-value-added OSA starch against the increased operational expenditures.

## 4. Conclusions

This study overcomes the limitations of the traditional batch addition method in the preparation of OSA-modified starch. It systematically elucidated the synergistic effects of OSA addition time, temperature, and total reaction time on the DS and physicochemical properties of NMS. Single-factor experiments revealed that optimal modification was achieved under the conditions of a 40 °C reaction temperature, a 2 h OSA addition time, and a 6 h total reaction time. The success of esterification was directly confirmed by FT-IR spectroscopy. The OSA-modified NMS exhibited two new absorption peaks at 1724 and 1572 cm^−1^ compared with the native NMS. This optimized process likely minimizes side reactions, such as competitive hydrolysis and alkaline degradation, by balancing starch granule swelling and reagent penetration. The modified starch prepared under these conditions exhibited superior functional properties, characterized by a significant reduction in gelatinization temperature and enthalpy. This is presumably attributed to the effective disruption of the starch crystalline structure facilitated by the deep penetration of OSA. Rheological evaluations indirectly corroborated these structural alterations: the modified starch displayed more pronounced pseudoplasticity, higher apparent viscosity, and superior anti-retrogradation properties. DS exhibited highly significant negative correlations with both Δ*H* and *G*′_max_ but was not related to pasting properties. Furthermore, a response surface methodology (RSM) model accurately predicted the theoretical optimal process parameters (temperature of 41.96 °C, OSA addition time of 2.25 h, and total reaction time of 6.9 h), yielding a verified DS value of 0.0258. Although macroscopic physicochemical data strongly support the hypothesis of different reaction locations in starch granules, this study has yet to incorporate direct microstructural characterizations. Consequently, future research could employ multi-scale techniques to provide more direct evidence. This research will provide more information for optimizing the industrial production process for food processors.

## Figures and Tables

**Table 1:** Experimental results of response surface methodology.

Trial	*A* (°C)	*B* (h)	*C* (h)	DS	
				Experimental	Predicted
1	45	2.5	6	0.0246	0.0246
2	45	1.5	6	0.0239	0.0237
3	35	2.5	6	0.0224	0.0226
4	35	1.5	6	0.0248	0.0248
5	45	2	8	0.0251	0.0252
6	45	2	4	0.0213	0.0214
7	35	2	8	0.0229	0.0228
8	35	2	4	0.0231	0.023
9	40	2.5	8	0.025	0.0249
10	40	2.5	4	0.0212	0.0211
11	40	1.5	8	0.0234	0.0235
12	40	1.5	4	0.0236	0.0237
13	40	2	6	0.0256	0.0257
14	40	2	6	0.0257	0.0257
15	40	2	6	0.0258	0.0257
16	40	2	6	0.0259	0.0257
17	40	2	6	0.0256	0.0257

**Table 2:** Pasting properties of OSA-modified NMS under varying single-factor conditions.

Factor	Condition	PV (mPa·s)	TV (mPa·s)	BD (mPa·s)	FV (mPa·s)	SB (mPa·s)
Temperature	Native	327 ± 6 ^e^	268 ± 6 ^c^	58 ± 1 ^d^	302 ± 6 ^d^	34 ± 1 ^d^
	0 °C	1630 ± 5 ^a^	1113 ± 41 ^b^	517 ± 37 ^a^	1617 ± 33 ^c^	504 ± 8 ^bc^
	25 °C	1350 ± 0 ^d^	1132 ± 20 ^b^	218 ± 20 ^c^	1665 ± 21 ^bc^	533 ± 1 ^a^
	35 °C	1469 ± 7 ^b^	1144 ± 39 ^b^	325 ± 36 ^b^	1653 ± 23 ^bc^	509 ± 19 ^b^
	40 °C	1404 ± 51 ^c^	1311 ± 56 ^a^	93 ± 13 ^d^	1798 ± 59 ^a^	487 ± 11 ^c^
	45 °C	1406 ± 9 ^c^	1150 ± 6 ^b^	256 ± 10 ^c^	1687 ± 7 ^b^	537 ± 11 ^a^
OSA addition time	Native	327 ± 6 ^e^	268 ± 6 ^d^	58 ± 1 ^d^	302 ± 6 ^d^	34 ± 1 ^c^
	0.5 h	1584 ± 20 ^a^	998 ± 22 ^bc^	587 ± 42 ^a^	1501 ± 13 ^b^	504 ± 35 ^a^
	1 h	1255 ± 55 ^c^	1008 ± 33 ^bc^	247 ± 31 ^b^	1380 ± 90 ^c^	372 ± 64 ^b^
	1.5 h	1160 ± 5 ^d^	1041 ± 14 ^b^	120 ± 19 ^c^	1452 ± 38 ^bc^	411 ± 51 ^b^
	2 h	1404 ± 51 ^b^	1311 ± 56 ^a^	93 ± 13 ^cd^	1798 ± 59 ^a^	487 ± 11 ^a^
	2.5 h	1201 ± 15 ^cd^	980 ± 20 ^c^	221 ± 13 ^b^	1495 ± 21 ^b^	515 ± 2 ^a^
Total reaction time	Native	327 ± 6 ^e^	268 ± 6 ^d^	58 ± 1 ^c^	302 ± 6 ^d^	34 ± 1 ^c^
	2 h	1236 ± 40 ^c^	906 ± 8 ^c^	330 ± 33 ^a^	1251 ± 71 ^c^	345 ± 64 ^b^
	4 h	1167 ± 8 ^d^	932 ± 50 ^c^	236 ± 42 ^b^	1294 ± 119 ^c^	363 ± 69 ^b^
	6 h	1404 ± 51 ^a^	1311 ± 56 ^a^	93 ± 13 ^c^	1798 ± 59 ^a^	487 ± 11 ^a^
	8 h	1299 ± 14 ^b^	1034 ± 52 ^b^	265 ± 56 ^ab^	1530 ± 17 ^b^	496 ± 37 ^a^

PV = Peak Viscosity, TV = Trough Viscosity, BD = Breakdown, FV = Final Viscosity, SB = Setback; Values are expressed as mean ± SD of triplicate measurements. Values in the same column within each individual single-factor group followed by different lowercase letters are significantly different (*p* < 0.05).

**Table 3:** Thermal properties of OSA-modified NMS under varying single-factor conditions.

Factor	Condition	*T*_o_ (°C)	*T*_p_ (°C)	*T*_c_ (°C)	∆*H* (J/g)
Temperature	Native	64.1 ± 0.1 ^a^	69.9 ± 0.1 ^a^	75.3 ± 0.2 ^a^	11.8 ± 0.3 ^a^
	0 °C	62.9 ± 0.1 ^b^	69.9 ± 0.1 ^a^	75.2 ± 0.1 ^ab^	10.3 ± 0.2 ^b^
	25 °C	62.4 ± 0.1 ^c^	69.7 ± 0.1 ^b^	75.2 ± 0.0 ^ab^	9.9 ± 0.2 ^c^
	35 °C	61.6 ± 0.1 ^d^	69.6 ± 0.1 ^bc^	75.1 ± 0.1 ^ab^	9.6 ± 0.1 ^d^
	40 °C	61.3 ± 0.1 ^e^	69.6 ± 0.1 ^c^	75.1 ± 0.0 ^b^	9.3 ± 0.1 ^d^
	45 °C	61.0 ± 0.1 ^f^	69.3 ± 0.1 ^d^	74.7 ± 0.1 ^c^	9.3 ± 0.1 ^d^
OSA addition time	Native	64.1 ± 0.1 ^a^	69.9 ± 0.1 ^a^	75.3 ± 0.2 ^a^	11.8 ± 0.3 ^a^
	0.5 h	62.9 ± 0.1 ^b^	69.9 ± 0.1 ^a^	75.2 ± 0.0 ^ab^	10.3 ± 0.1 ^b^
	1 h	62.3 ± 0.2 ^c^	69.8 ± 0.1 ^ab^	75.2 ± 0.1 ^ab^	9.8 ± 0.2 ^c^
	1.5 h	61.3 ± 0.2 ^e^	69.7 ± 0.1 ^cd^	75.1 ± 0.0 ^b^	9.5 ± 0.1 ^d^
	2 h	61.3 ± 0.1 ^e^	69.6 ± 0.1 ^d^	75.1 ± 0.0 ^b^	9.3 ± 0.1 ^d^
	2.5 h	61.5 ± 0.1 ^d^	69.7 ± 0.1 ^bc^	75.2 ± 0.1 ^ab^	9.5 ± 0.1 ^d^
Total reaction time	Native	64.1 ± 0.1 ^a^	69.9 ± 0.1 ^b^	75.3 ± 0.2 ^ab^	11.8 ± 0.3 ^a^
	2 h	63.3 ± 0.1 ^b^	70.0 ± 0.1 ^a^	75.3 ± 0.1 ^a^	10.4 ± 0.0 ^b^
	4 h	62.3 ± 0.0 ^c^	69.7 ± 0.1 ^c^	75.2 ± 0.1 ^ab^	9.8 ± 0.1 ^c^
	6 h	61.3 ± 0.1 ^e^	69.6 ± 0.1 ^d^	75.1 ± 0.0 ^b^	9.3 ± 0.1 ^d^
	8 h	61.4 ± 0.1 ^d^	69.6 ± 0.0 ^cd^	75.1 ± 0.1 ^ab^	9.4 ± 0.2 ^d^

*T*_o_ = onset temperature, *T*_p_ = peak temperature, *T*_c_ = conclusion temperature, and ∆*H* = enthalpy change; Values are expressed as mean ± SD of triplicate measurements. Values in the same column within each individual single-factor group followed by different lowercase letters are significantly different (*p* < 0.05).

**Table 4:** Power Law parameters of OSA-modified NMS under varying single-factor conditions.

Factor	Condition	Upward Curve			Downward Curve		
		*K* (Pa·s^*n*^)	*n*	*R* ^2^	*K* (Pa·s^*n*^)	*n*	*R* ^2^
Temperature	Native	7.3 ± 0.0 ^f^	0.375 ± 0.003 ^a^	0.99	7.1 ± 0.0 ^f^	0.386 ± 0.001 ^d^	0.99
	0 °C	30.5 ± 0.4 ^e^	0.336 ± 0.006 ^d^	0.99	19.2 ± 0.3 ^e^	0.414 ± 0.002 ^a^	0.99
	25 °C	33.8 ± 0.1 ^d^	0.346 ± 0.003 ^c^	0.99	24.0 ± 0.2 ^d^	0.406 ± 0.003 ^b^	0.99
	35 °C	34.2 ± 0.2 ^c^	0.347 ± 0.004 ^c^	0.99	25.5 ± 0.3 ^c^	0.399 ± 0.004 ^c^	0.99
	40 °C	36.1 ± 0.3 ^a^	0.353 ± 0.005 ^b^	0.99	27.6 ± 0.2 ^a^	0.397 ± 0.006 ^c^	0.99
	45 °C	34.9 ± 0.2 ^b^	0.350 ± 0.005 ^bc^	0.99	26.6 ± 0.2 ^b^	0.400 ± 0.006 ^c^	0.99
OSA addition time	Native	7.3 ± 0.0 ^f^	0.375 ± 0.003 ^a^	0.99	7.1 ± 0.0 ^f^	0.386 ± 0.001 ^d^	0.99
	0.5 h	30.5 ± 0.2 ^e^	0.336 ± 0.002 ^d^	0.99	18.3 ± 0.1 ^e^	0.409 ± 0.001 ^a^	0.99
	1 h	33.2 ± 0.3 ^d^	0.347 ± 0.002 ^c^	0.99	24.1 ± 0.2 ^d^	0.402 ± 0.003 ^b^	0.99
	1.5 h	35.7 ± 0.3 ^b^	0.353 ± 0.001 ^b^	0.99	27.1 ± 0.2 ^b^	0.396 ± 0.002 ^c^	0.99
	2 h	36.1 ± 0.3 ^a^	0.353 ± 0.005 ^b^	0.99	27.6 ± 0.2 ^a^	0.397 ± 0.006 ^c^	0.99
	2.5 h	35.0 ± 0.4 ^c^	0.348 ± 0.003 ^c^	0.99	25.8 ± 0.3 ^c^	0.400 ± 0.002 ^bc^	0.99
Total reaction time	Native	7.3 ± 0.0 ^d^	0.375 ± 0.003 ^a^	0.99	7.1 ± 0.0 ^e^	0.386 ± 0.001 ^d^	0.99
	2 h	27.2 ± 0.3 ^c^	0.319 ± 0.004 ^d^	0.99	16.6 ± 0.2 ^d^	0.415 ± 0.004 ^a^	0.99
	4 h	33.1 ± 0.5 ^b^	0.341 ± 0.010 ^c^	0.99	23.6 ± 0.4 ^c^	0.405 ± 0.002 ^b^	0.99
	6 h	36.1 ± 0.3 ^a^	0.353 ± 0.005 ^b^	0.99	27.6 ± 0.2 ^a^	0.397 ± 0.006 ^c^	0.99
	8 h	35.8 ± 0.1 ^a^	0.351 ± 0.001 ^b^	0.99	26.9 ± 0.1 ^b^	0.405 ± 0.009 ^bc^	0.99

Values are expressed as mean ± SD of triplicate measurements. Values in the same column within each individual single-factor group followed by different lowercase letters are significantly different (*p* < 0.05).

**Table 5:** Dynamic oscillatory properties of OSA-modified NMS under varying single-factor conditions.

Factor	Condition	Heating Process				95 °C			Cooling Process		
		*G*′_max_ (Pa)	*G*″_*G*′max_ (Pa)	tan *δ*_*G*′max_	*T*_*G*′max_ (°C)	*G*′_95°C_ (Pa)	*G*″_95°C_ (Pa)	tan *δ*_95°C_	*G*′_25°C_ (Pa)	*G*″_25°C_ (Pa)	tan *δ*_25°C_
Temperature	Native	4933 ± 61 ^a^	470 ± 8 ^c^	0.095 ± 0.000 ^f^	78.6 ± 0.0 ^a^	2302 ± 81 ^a^	213 ± 5 ^a^	0.093 ± 0.001 ^c^	6542 ± 29 ^a^	190 ± 4 ^a^	0.029 ± 0.000 ^e^
	0 °C	3269 ± 18 ^b^	567 ± 4 ^b^	0.173 ± 0.000 ^e^	75.0 ± 0.0 ^b^	1334 ± 35 ^b^	174 ± 3 ^c^	0.130 ± 0.001 ^b^	2212 ± 33 ^b^	132 ± 5 ^c^	0.059 ± 0.001 ^d^
	25 °C	3042 ± 18 ^c^	585 ± 2 ^ab^	0.192 ± 0.001 ^d^	74.7 ± 0.0 ^c^	1284 ± 12 ^b^	180 ± 2 ^bc^	0.140 ± 0.002 ^b^	2036 ± 15 ^c^	136 ± 7 ^bc^	0.067 ± 0.003 ^c^
	35 °C	2881 ± 36 ^d^	584 ± 8 ^ab^	0.203 ± 0.000 ^c^	74.6 ± 0.0 ^c^	1223 ± 33 ^bc^	187 ± 6 ^bc^	0.153 ± 0.001 ^a^	1920 ± 47 ^cd^	139 ± 7 ^bc^	0.072 ± 0.002 ^bc^
	40 °C	2725 ± 46 ^e^	604 ± 15 ^a^	0.222 ± 0.002 ^a^	74.3 ± 0.1 ^d^	1189 ± 49 ^bc^	191 ± 8 ^b^	0.160 ± 0.000 ^a^	1899 ± 66 ^d^	153 ± 4 ^b^	0.080 ± 0.001 ^a^
	45 °C	2929 ± 56 ^cd^	606 ± 9 ^a^	0.207 ± 0.001 ^b^	74.3 ± 0.0 ^d^	1107 ± 38 ^c^	173 ± 3 ^c^	0.156 ± 0.008 ^a^	1861 ± 27 ^d^	140 ± 5 ^bc^	0.075 ± 0.002 ^ab^
OSA addition time	Native	4933 ± 61 ^a^	470 ± 8 ^c^	0.095 ± 0.000 ^f^	78.6 ± 0.0 ^a^	2302 ± 81 ^a^	213 ± 5 ^a^	0.093 ± 0.001 ^d^	6542 ± 29 ^a^	190 ± 4 ^a^	0.029 ± 0.000 ^d^
	0.5 h	3329 ± 40 ^b^	573 ± 9 ^b^	0.172 ± 0.001 ^e^	75.2 ± 0.0 ^b^	1400 ± 21 ^b^	172 ± 8 ^b^	0.123 ± 0.004 ^c^	2396 ± 28 ^b^	139 ± 3 ^c^	0.058 ± 0.002 ^c^
	1 h	3115 ± 30 ^c^	591 ± 2 ^ab^	0.190 ± 0.001 ^d^	74.7 ± 0.0 ^c^	1248 ± 14 ^c^	180 ± 2 ^b^	0.144 ± 0.003 ^b^	2067 ± 11 ^c^	149 ± 5 ^bc^	0.072 ± 0.002 ^b^
	1.5 h	2826 ± 8 ^de^	609 ± 1 ^a^	0.215 ± 0.000 ^b^	74.6 ± 0.1 ^d^	1105 ± 18 ^d^	177 ± 6 ^b^	0.160 ± 0.002 ^a^	1841 ± 4 ^d^	151 ± 1 ^b^	0.082 ± 0.000 ^a^
	2 h	2725 ± 46 ^e^	604 ± 15 ^a^	0.222 ± 0.002 ^a^	74.3 ± 0.1 ^e^	1189 ± 49 ^cd^	191 ± 8 ^b^	0.160 ± 0.000 ^a^	1899 ± 66 ^d^	153 ± 4 ^b^	0.080 ± 0.001 ^a^
	2.5 h	2878 ± 33 ^d^	598 ± 11 ^ab^	0.208 ± 0.001 ^c^	74.6 ± 0.0 ^d^	1203 ± 32 ^cd^	187 ± 6 ^b^	0.155 ± 0.001 ^a^	1930 ± 7 ^d^	150 ± 4 ^bc^	0.078 ± 0.002 ^a^
Total reaction time	Native	4933 ± 61 ^a^	470 ± 8 ^c^	0.095 ± 0.000 ^e^	78.6 ± 0.0 ^a^	2302 ± 81 ^a^	213 ± 5 ^a^	0.093 ± 0.001 ^d^	6542 ± 29 ^a^	190 ± 4 ^a^	0.029 ± 0.000 ^d^
	2 h	3370 ± 49 ^b^	551 ± 8 ^b^	0.164 ± 0.000 ^d^	75.2 ± 0.1 ^b^	1432 ± 9 ^b^	172 ± 3 ^b^	0.120 ± 0.001 ^c^	2515 ± 34 ^b^	151 ± 8 ^b^	0.060 ± 0.002 ^c^
	4 h	3054 ± 36 ^c^	581 ± 3 ^a^	0.190 ± 0.001 ^c^	74.6 ± 0.1 ^c^	1309 ± 49 ^bc^	187 ± 8 ^b^	0.142 ± 0.000 ^b^	2012 ± 26 ^c^	140 ± 3 ^b^	0.069 ± 0.000 ^b^
	6 h	2725 ± 46 ^d^	604 ± 15 ^a^	0.222 ± 0.002 ^b^	74.3 ± 0.1 ^d^	1189 ± 49 ^cd^	191 ± 8 ^b^	0.160 ± 0.000 ^a^	1899 ± 66 ^cd^	153 ± 4 ^b^	0.080 ± 0.001 ^a^
	8 h	2843 ± 8 ^d^	599 ± 4 ^a^	0.211 ± 0.002 ^a^	74.4 ± 0.0 ^d^	1122 ± 18 ^d^	179 ± 3 ^b^	0.159 ± 0.000 ^a^	1874 ± 29 ^d^	148 ± 3 ^b^	0.079 ± 0.003 ^a^

Values are expressed as mean ± SD of triplicate measurements. Values in the same column within each individual single-factor group followed by different lowercase letters are significantly different (*p* < 0.05).

**Table 6:** Pearson correlation coefficients among DS and physicochemical properties.

	DS	PV	FV	Δ*H*	*G*′_max_
DS	—	−0.307 ^ns^	+0.503 ^ns^	−0.960 ***	−0.972 ***
PV		—	+0.577 *	+0.337 ^ns^	+0.319 ^ns^
FV			—	−0.457 ^ns^	−0.504 ^ns^
Δ*H*				—	+0.957 ***
*G*′_max_					—

^ns^: not significant; *: *p* < 0.05; ***: *p* < 0.001.

**Table 7:** Simple linear regression analysis between DS and physicochemical properties.

*Y* Variable	Pearson *r*	*p*-Value	*R* ^2^	Slope	Intercept	Sig.
PV (mPa·s)	−0.307	0.3319	0.094	−20,856	+1831	ns
FV (mPa·s)	+0.503	0.0953	0.253	+36,160	+687	ns
Δ*H* (J/g)	−0.960	<0.0001	0.922	−165.2	+13.59	***
*G*′_max_ (Pa)	−0.972	<0.0001	0.945	−89,449	+5099	***

Regression model: *Y* = Slope × DS + Intercept; ns: not significant; ***: *p* < 0.001.

**Table 8:** Estimated regression model for relationship between response variables (DS) and independent variables (*A*, *B*, *C*).

Source	Sum of Squares	Degree of Freedom	Mean Square	*F*-Value	*p*-Value
Model	3.78 × 10^−5^	9	4.20 × 10^−6^	108.77	<0.0001
*A*	3.61 × 10^−7^	1	3.61 × 10^−7^	9.35	0.0184
*B*	7.81 × 10^−7^	1	7.81 × 10^−7^	20.22	0.0028
*C*	6.48 × 10^−6^	1	6.48 × 10^−6^	167.69	<0.0001
*AB*	2.40 × 10^−6^	1	2.40 × 10^−6^	62.17	<0.0001
*AC*	4.00 × 10^−6^	1	4.00 × 10^−6^	103.51	<0.0001
*BC*	4.00 × 10^−6^	1	4.00 × 10^−6^	103.51	<0.0001
*A* ^2^	4.19 × 10^−6^	1	4.19 × 10^−6^	108.42	<0.0001
*B* ^2^	2.68 × 10^−6^	1	2.68 × 10^−6^	69.30	<0.0001
*C* ^2^	1.11 × 10^−5^	1	1.11 × 10^−5^	286.84	<0.0001
Lack of Fit	2.03 × 10^−7^	3	6.75 × 10^−8^	3.97	0.1080
Adjusted *R*^2^	0.9838				
Predicted *R*^2^	0.9122				
Adequate Precision	30.7259				
CV (%)	0.8153				

## Data Availability

The original contributions presented in this study are included in the article. Further inquiries can be directed to the corresponding author.
